# Skin on Drugs: Psychotropic Compounds in Cutaneous Biology

**DOI:** 10.3390/ijms27135808

**Published:** 2026-06-26

**Authors:** Montserrat Fernández-Guarino, Nicolás Yagüe-Septién, Laura Marín-Ochoa, María Luisa Hernández Bule, Stefano Bacci, Ana Banzo, Daniel Peña-Jiménez

**Affiliations:** 1 Dermatology Service, Hospital Universitario Ramon y Cajal, 28034 Madrid, Spain; 2 Grupo de Investigación de Fotobiología, Terapias con Fuentes de Luz y Prevención de Cáncer Universidad Alfonso X el Sabio (UAX), Avenida de la Universidad 1, Villanueva de la Cañada, 28691 Madrid, Spain; stefano.bacci@unifi.it; 3 Unidad de Investigación Biomédica, Universidad Alfonso X el Sabio (UAX), Avenida de la Universidad 1, Villanueva de la Cañada, 28691 Madrid, Spain; nyagusep@myuax.com (N.Y.-S.); lmarioch@uax.es (L.M.-O.);; 4 Department of Preclinical Dentistry I, Faculty of Biomedical and Health Sciences, Universidad Europea de Madrid, 28670 Madrid, Spain; marialuisa.hernandez@universidadeuropea.es; 5 Research Unit of Histology and Embriology, Department of Biology, University of Florence, Viale Pieraccini 6, 50134 Firenze, Italy

**Keywords:** skin, cannabinoids, psychedelics, antidepressants, inflammation

## Abstract

Recent evidence reveals that several psychotropic compounds exert significant biological effects on the skin through neurochemical and immunomodulatory pathways. Cannabinoids such as tetrahydrocannabinol (THC) show potent anti-inflammatory, antipruritic, and anti-aging properties when applied topically, and may hold therapeutic potential. Antidepressants, particularly fluoxetine (Prozac), have been shown to regulate the expression of pro-inflammatory cytokines in keratinocytes, suggesting benefits applied in allergic pathologies. Additionally, fluoxetine promotes wound healing and cell regeneration, indicating broader dermatological applications. Psychedelics, acting as serotonin receptor agonists (5-HTR), may influence cellular aging and immune modulation via the serotonergic system. Studies report that 5-HT receptor agonists can prevent UV-induced photocarcinogenesis, while psilocybin has been observed to reduce aging markers in human fibroblasts. Furthermore, recent data suggests that psilocin may alleviate acute itch involving the kynurenine pathway. These findings highlight the emerging relevance of psychoactive compounds in cutaneous biology, bridging neuropharmacology and dermatology toward novel therapeutic strategies.

## 1. Introduction

The skin, traditionally regarded as a passive physical barrier, is now recognized as a highly dynamic neuro–endocrine–immune organ that actively integrates environmental, immunological, and neurochemical signals to maintain tissue homeostasis [1,2,3,4]. Beyond its structural and protective roles, the skin is equipped with a wide range of receptors for neurotransmitters, hormones, and other neuromodulators, allowing it to actively communicate with both the nervous and immune systems. This growing understanding has changed how we view skin biology, highlighting the importance of neuroimmune and neuroendocrine interactions as key regulators of skin function and disease.

In this context, psychotropic compounds, historically developed and used for central nervous system disorders, have emerged as unexpected modulators of peripheral tissues, including the skin. Cannabinoids, antidepressants, and serotonergic psychedelics have been shown to interact with cutaneous signaling networks, influencing key biological processes such as inflammation, pruritus, wound healing, barrier function, and cellular aging. For instance, the cutaneous endocannabinoid system (ECS) plays a pivotal role in epidermal homeostasis and immune regulation, and its pharmacological modulation has demonstrated therapeutic potential in inflammatory and barrier-related skin disorders [5,6,7]. Similarly, antidepressants, particularly selective serotonin reuptake inhibitors (SSRIs), exhibit clinically relevant antipruritic and immunomodulatory effects, although their broader dermatological applications remain under investigation [8,9,10]. More recently, serotonergic psychedelics have gained attention for their capacity to modulate immune responses and cellular senescence pathways, suggesting a novel role in regenerative and anti-aging dermatology [11,12].

Despite these advances, the field remains fragmented and, in some aspects, controversial. While cannabinoids are supported by a growing body of translational and early clinical evidence, data on psychedelics are predominantly preclinical, and their dermatological relevance is still speculative. In parallel, antidepressants present a dual profile, with beneficial immunomodulatory effects on one hand and potential pro-inflammatory or mast cell-activating properties on the other, highlighting the context-dependent nature of their actions [13,14]. Furthermore, significant challenges persist regarding drug delivery, skin penetration, compound stability, and regulatory constraints, which limit the clinical translation of these compounds into dermatological therapies.

In this review, we provide a comprehensive and integrative overview of the effects of major classes of psychotropic compounds on cutaneous biology. We aim to dissect their underlying molecular mechanisms, evaluate their impact on key skin processes, and critically assess their current and potential therapeutic applications. Overall, the available evidence supports a growing role for psychotropic agents as modulators of skin homeostasis, although most data remain preclinical and further studies are required to validate their clinical utility. This emerging intersection between neuropharmacology and dermatology may ultimately lead to the development of innovative therapeutic strategies targeting skin diseases. The main classes of psychotropic compounds and their molecular targets and cutaneous effects are summarized in Table 1.

## 2. The Skin

### 2.1. General Morphological Characteristics of the Skin

**Figure 1:**
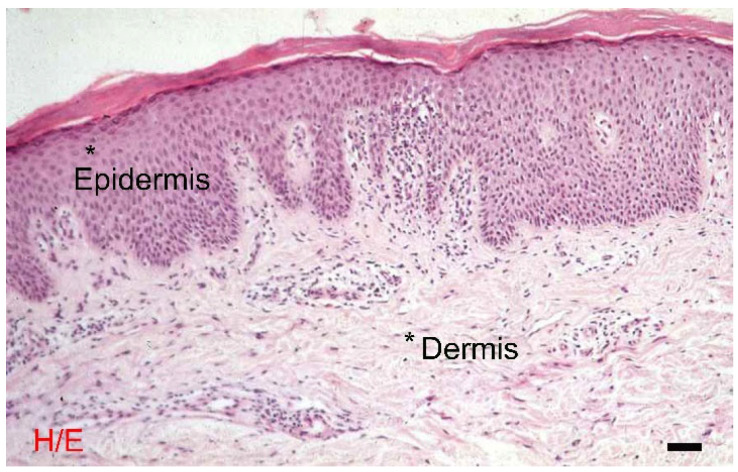
The skin: Morphological differences between the epidermis and the dermis. Light microscopy, hematoxylin and eosin, scale bar = 100 μm [15]. * Epidermis, * Dermis.

The skin, or cutis, along with the subcutaneous connective tissue, forms the body’s integument, covering the entire external surface and transitioning to mucous membranes. It consists of an epidermis (surface epithelial tissue) and a dermis (underlying connective tissue), with subcutaneous fat contributing to its structure. The skin accounts for approximately 5–6% of body weight, with surface area related to height and weight; various estimation methods exist, including a division into 11 districts for injury assessment. Skin thickness is variable, ranging from 0.5 mm to 4 mm depending on body location, with epidermis thickness varying from 50 to 150 µm generally, and up to 1.5 mm in thicker areas like palms and soles. Surface features include skin ridges and furrows, while aging and sun exposure can cause wrinkles. The skin exhibits distensibility and elasticity, with surgical incisions needing alignment with the direction of greatest tension to minimize scar diastasis (Figure 1) [15,16,17,18,19].

The skin serves multiple protective functions against physical, chemical, and biological threats, as well as regulating water loss and heat dispersion. The epidermis provides a flexible and impermeable barrier while housing melanin for UV protection. The dermis offers mechanical support with connective fibers, and the subcutaneous layer cushions impacts and maintains thermal insulation. Additionally, the skin plays a vital role in communication and sensory perception, facilitating interactions and stimuli recognition. The subcutaneous tissue also acts as a nutritional reserve and hormone source, influencing various metabolic processes [15,16,17,18,19].

### 2.2. The Epidermis

**Figure 2:**
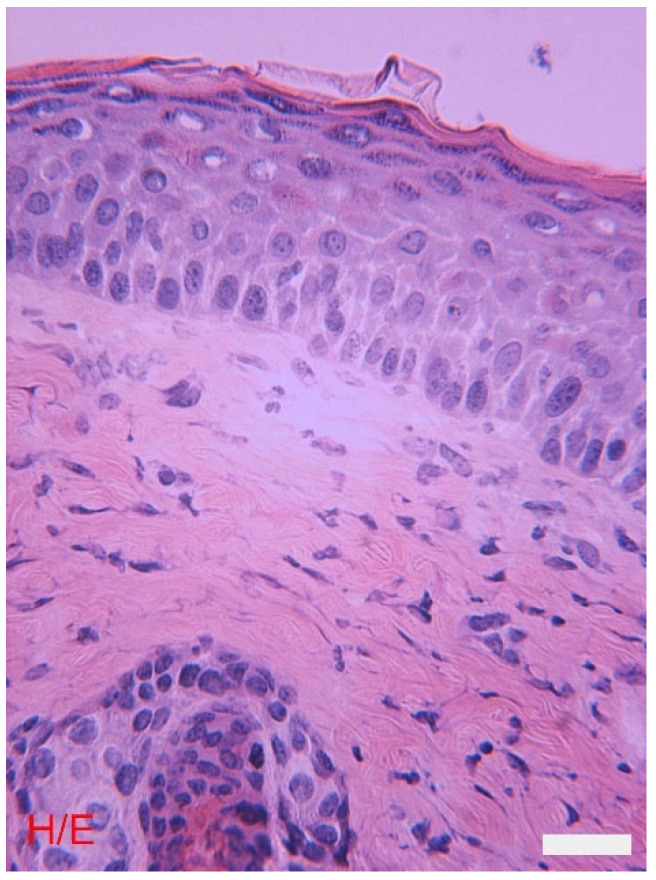
The skin: Morphological differences between the epidermis and the dermis. In the epidermis the various layers that compose it. The underlying dermis has various cell types, a sebaceous gland and a hair follicle. Light microscopy, hematoxylin and eosin, scale bar = 10 μm [15].

The epidermis is a keratinized compound epithelium composed of several layers, namely the basal, spinous, granular, and corneous layers, with the stratum lucidum identifiable in thick skin (Figure 2). Primarily consisting of keratinocytes, which differentiate into stratum corneum cells, the epidermis also contains around 10–15% of other cell types, including melanocytes, Langerhans cells, Merkel cells, and lymphocytes, each with distinct functions [15,16,17,18,19].

The term keratin refers to the structural proteins within the epidermis, particularly in the outermost layer, the stratum corneum. Keratin exists as filamentous molecules, with variations found in deeper epidermal layers as tonofibrils and tonofilaments, which serve as intermediate filaments for keratinocytes. Cytokeratins, a family of proteins, are critical in forming these structures. The basal layer is characterized by cells’ capacity to reproduce, with certain stem cells maintaining lifelong proliferative ability. Melanocytes, which produce melanin, have a key role in skin pigmentation and are distributed throughout the epidermis, affecting skin color regardless of their density. The skin’s normal color is influenced by melanin, hemoglobin, and the stratum corneum’s properties, along with environmental light conditions. Merkel cells within the basal layer are involved in tactile sensations. The spinous layer consists of large polyhedral cells interconnected by desmosomes, with abundant tonofibrils. The stratum granulosum contains keratohyalin granules contributing to the outer layer’s formation, and its cells have occluding bands enhancing impermeability. Finally, the stratum corneum comprises flattened, dehydrated corneocytes with a unique membrane and lipid composition, crucial for maintaining skin barrier functions. In thick skin regions, the stratum lucidum is present, characterized by the eleidin granules, serving to create a transparent appearance when layers are removed [15,16,17,18,19].

### 2.3. The Dermis

The dermis, located beneath the epidermis, consists of dense fibrous connective tissue, divided into the papillary dermis (superficial portion with fine fiber bundles) and the reticular dermis (deeper with coarse fiber bundles). To localize structures and potential alterations, the dermis is often categorized into superficial, medium, and deep layers, with the superficial layer corresponding to the papillary dermis and the others referencing levels within the reticular dermis [15,16,17,18,19].

**Figure 3:**
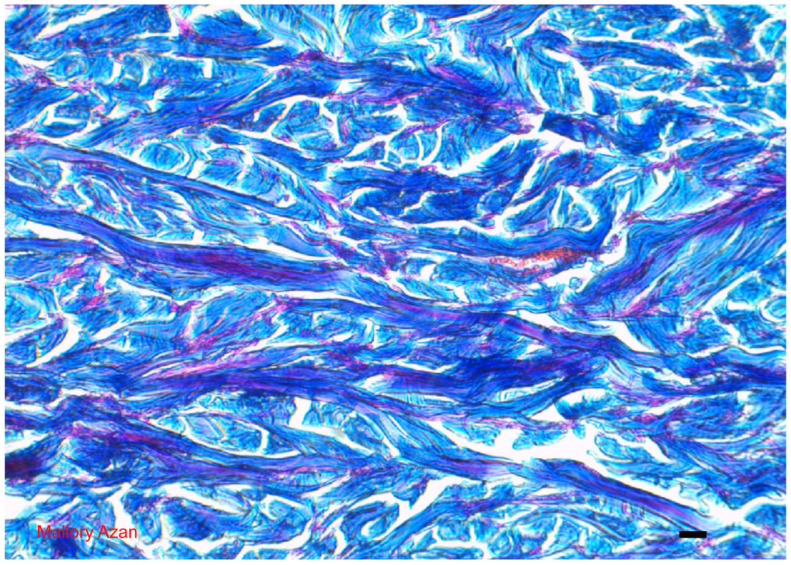
Dermis: Different features of the collagen fibers which histologically define the type of tissue considered. Light microscopy, Mallory Azan, scale bar = 10 μm [15].

The dermis consists of a rich extracellular matrix made up of fibers, predominantly collagen type I, and an amorphous ground substance (Figure 3). Collagen fibers are polymers of tropocollagen, arranged in various orientations, forming fibrils that are visible under an electron microscope. These fibrils, stabilized by covalent bonds over several weeks, become insoluble and are cemented together in a fiber structure. The dermis’s resistance to tension is due to the inextensibility of collagen fibers, while its deformability relies on the fibers’ ability to realign and straighten under stress [15,16,17,18,19].

The elastic fibers in the dermis consist of tubular microfibrils made of fibrillin, which are inextensible, and an amorphous matrix primarily composed of elastin, providing elasticity. These fibers maintain a degree of basic tension, varying by direction, influencing wound shape during healing and surgical cuts to minimize tension on stitches. The amorphous fundamental substance consists mainly of water and contains glycoproteins and proteoglycans, which facilitate cellular function and water diffusion. This substance also helps in maintaining dermal turgor, resistance, and elasticity against compression [15,16,17,18,19].

The dermis is rich in fibroblasts and macrophages. Fibroblasts synthesize the extracellular matrix, organizing collagen and elastic fibers, with various enzymes essential for collagen maturation and stabilization. Congenital enzyme defects can lead to collagen deficiencies, causing dermal laxity and fragility. Myofibroblasts, specialized fibroblasts, contract and facilitate dermal healing, but their persistence in tissues is still debated. Macrophages play a critical role in removing foreign material and repairing tissue damage [15,16,17,18,19].

The basement membrane is a specialized layer of the extracellular matrix located just beneath the epidermis and skin appendages, observable through silver impregnations. It consists of three layers: the lamina rare (20–30 nm thick), which serves as the glycocalyx of epithelial cells, the lamina dense (20–50 nm thick), a mixture of type IV collagen and glycoproteins like laminin, and the reticular lamina, which is thicker and connects to the dermis. The lamina dense is the most characteristic portion of the basement membrane, while the reticular lamina contains reticular fibers, anchoring fibrils, and oxythalanic fibers. The anchoring fibrils, made of type VII collagen, are exclusive to the basement membrane. This structure facilitates the adhesion of epithelial cells through hemidesmosomes and acts as a barrier against the diffusion of large molecules. Additionally, the basement membrane provides crucial signals to adjacent cells, influencing their differentiation from the basal layer of the epithelium [15,16,17,18,19].

### 2.4. The Subcutaneous

In the hypodermis, adipose tissue forms lobules divided by retinacula connecting the dermis to the muscular fascia or periosteum. The subcutaneous tissue consists of a superficial areolar layer with rounded lobules and a deeper lamellar layer that is flattened and fewer in cells, facilitating movement of the skin. The deep layer is absent in the palms and soles, causing a strong attachment of the dermis to muscle fascia. In areas like the head, neck, and proximal thigh, superficial fascia separates the two layers, with fur muscles developing in the superficial fascia. Subcutaneous bags can form within the deep layer [15,16,17,18,19].

### 2.5. Vascularization and Innervation of the Skin

Arteries penetrate the skin via subcutaneous retinacula, creating a deep plexus in the dermis that branches into a subpapillary plexus, supplying vessels near the epidermis. Veins mirror this structure with four dermal plexuses, while lymphatics form two: subpapillary and deep. The papillary capillary bed is highly developed, servicing primarily circulation for thermoregulation rather than metabolic needs, utilizing arteriovenous anastomoses, known as glomas, to bypass cooling when necessary [15,20,21].

**Figure 4:**
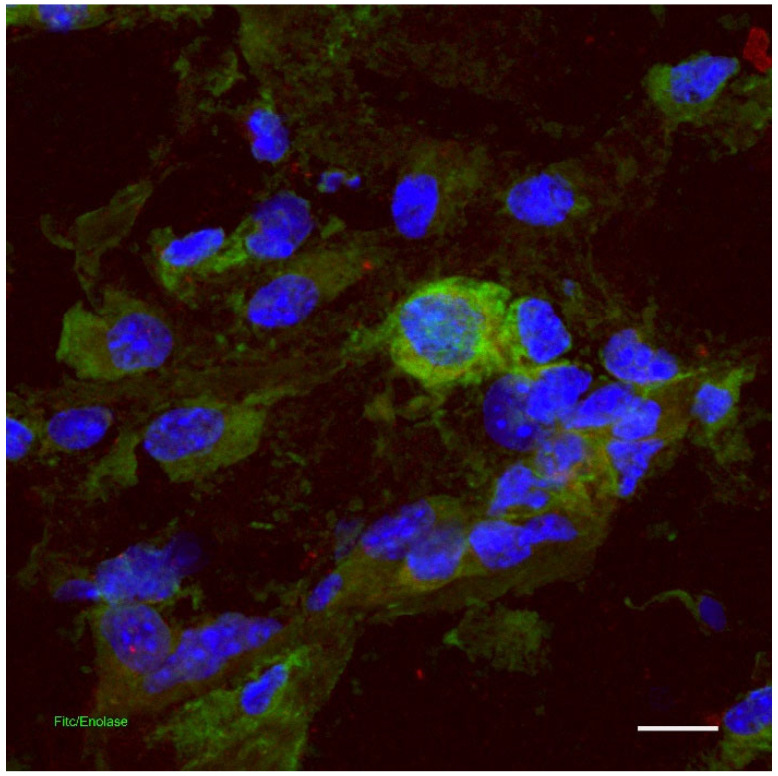
Dermis: Indirect immunofluorescence for the detection of neuronal enolase (green) in the skin and counterstained with DAPI (blue). Confocal microscopy, scale bar = 100 μm [15].

The skin contains a complex network of nerves, with both efferent fibers connecting to vessels and glands and afferent fibers associated with various sensory receptors (Figure 4). These receptors include free nerve endings in the epidermis and encapsulated structures like Meissner corpuscles, Pacinian corpuscles, and Ruffini corpuscles located in the dermis, each designed to respond to different tactile stimuli such as surface pressure, vibrations, and tissue distension. Additionally, hair follicles (HFs) are surrounded by nerve endings that contribute to tactile sensitivity, with the overall innervation allowing for tactile, thermal, and pain sensation in the skin [15,20,21].

### 2.6. Skin Immune System

**Figure 5:**
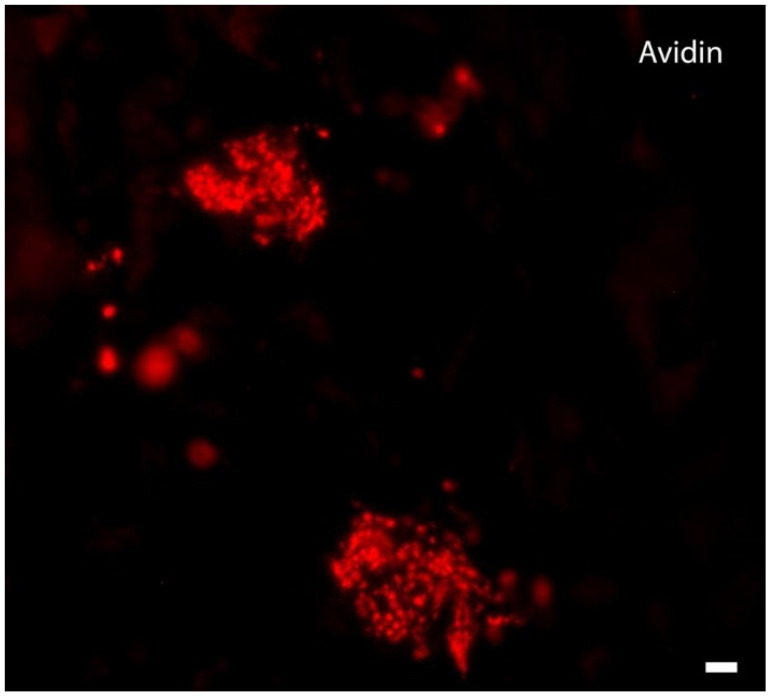
Mast cells scattered in the dermis. Fluorescence microscopy, scale bar = 10 μm.

The skin serves as a protective barrier against external threats, equipped with a defense system against infections and tumors promoted by environmental carcinogens, such as UV radiation. Its immune response involves the entire epidermis, skin appendages, and neural components, which collectively influence skin health and defense. A specialized group of cells, known as the cutaneous immune system, plays a crucial role in this defense. Dendritic cells in both the epidermis and dermis are essential for antigen presentation, capable of activating T lymphocytes and initiating immune responses by exposing epitopes to these cells. They are crucial for both primary and secondary immune responses, transitioning from antigen absorption to the ability to present these antigens to lymphocytes as they travel to the lymph nodes or spleen. Their development from immature to mature dendritic cells signifies their role in effectively stimulating the immune system. Dendritic cells, found in the epidermis and dermis, are specialized antigen-presenting cells with a branched shape. They play a crucial role in the immune response by absorbing antigens, breaking them down into epitopes, and presenting them along with accessory molecules to T lymphocytes. Dendritic cells can activate both helper T lymphocytes, leading to antibody secretion, and cytotoxic T lymphocytes, which defend against viruses and tumors. They utilize class II histocompatibility molecules for helper T cells and class I for cytotoxic T cells. Additionally, dendritic cells display costimulatory molecules required for initiating responses in naïve T lymphocytes. Essential for both primary and secondary immune responses, dendritic cells migrate to lymph nodes or the spleen during primary responses, while in secondary responses, other cells like macrophages also present antigens. They originate from monocyte precursors and differentiate into immature dendritic cells, further maturing as they reach lymphoid organs, ultimately specializing in antigen presentation rather than absorption. The epidermis contains Langerhans cells, specialized dendritic cells located primarily in the basal and spinous layers. They interact with keratinocytes through E-cadherin and contain Birbeck granules, which are involved in antigen processing. Langerhans cells have a slow renewal rate, exceeding 18 months, but this can decrease after stimuli like UV radiation. In the dermis, dendritic cells present different characteristics and renew more quickly, having roles similar to macrophages, which also participate in immune responses. Macrophages engulf foreign substances and can present antigens. Mast cells, containing histamine, heparin, and Tumor Necrosis Factor-α (TNF-α), respond to irritants and immune stimuli, secreting granules and active derivatives that affect other immune cells and tissue repair (Figure 5). Excessive mast cell activation can lead to allergic reactions due to excessive histamine release [15,20,21,22].

### 2.7. The Skin as a Neuroendocrine Organ

The interplay between human skin and the endocrine system has evolved from a traditional endocrinological lens, which largely viewed the skin as a passive recipient of hormonal signals, into a more dynamic understanding proposing that the skin itself functions as an active neuroendocrine organ. Historically examined primarily by endocrinologists, the endocrine influences on skin have focused on how systemic hormones such as cortisol, sex hormones, thyroid hormones, adrenocorticotropic hormone (ACTH), and growth hormone (GH) induce distinct dermatological manifestations when dysregulated. However, recent studies illustrate that human skin expresses a plethora of receptors for these neurohormones and is capable of synthesizing and secreting its own neuroendocrine factors, suggesting a more autonomous role in its physiological regulation [1,2,3,4].

Indeed, the past two decades have witnessed a paradigm shift, highlighting the skin’s capacity to produce various neurohormones and utilize them in biological processes, challenging the long-standing perception of skin as merely a target for external endocrine signals. Microanatomical examinations have revealed significant expression of neurohormones such as corticotropin-releasing hormone (CRH) and thyrotropin-releasing hormone (TRH) within skin structures, including HFs, which elude detection in serum but play crucial roles in local physiological responses [1,2,3,4].

The neuroendocrinology of the skin thus refers to both the skin’s ability to generate and respond to classical neurohormones and explore the vast array of biological activities facilitated by specific receptors. This recontextualization has significant clinical implications, potentially transforming therapeutic strategies for various skin disorders, including wound healing, hair growth disorders, chronic inflammatory conditions, and rare genetic skin diseases [1,2,3,4].

The evolutionary context of this complexity hints at a primitive origin of neuromediators, perhaps first arising in early skin-like epithelial structures, which governed fundamental biological responses long before the development of sophisticated neuroendocrine systems in vertebrates. Notably, certain hormones and neuropeptides, first identified in amphibian skin rather than neuronal tissues, indicate that skin may have originally served neuroendocrine functions [1,2,3,4].

Further insights indicate that both TRH and Thyroid-Stimulating Hormone (TSH) notably enhance mitochondrial function in the human epidermis and HFs, suggesting an intricate relationship between neuroendocrine signaling and cellular metabolism that had previously been underappreciated. Moreover, evidence suggests the existence of a local neuroendocrine axis in human skin similar to the central hypothalamus–pituitary–thyroid (HPT) axis, with epidermal production of TSH regulated analogously to traditional endocrine pathways [1,2,3,4].

The study of neurohormone-induced skin conditions potentially offers a window into understanding conserved ancestral functions, helping to elucidate complex skin phenotypes correlated with neurohormonal dysfunction. This involves a detailed analysis of skin manifestations linked with hormonal abnormalities, such as alopecia, hyperpigmentation, and inflammatory skin conditions, underscoring the need for rigorous exploration of the molecular mechanisms involved [1,2,3,4].

The skin, serving as the largest organ in the body, is integral not only to barrier and immune functions but also regulates fluid balance, temperature, and metabolic activities. It incorporates a diverse array of neuroendocrine factors produced within its layers, including classical neurohormones and lipid mediators, which collectively influence homeostasis [1,2,3,4]. Hormonal synthesis in skin occurs via activation of circulating precursors and de novo production, exemplified by the conversion of prohormones and steroid precursors directly within keratinocytes. This local synthesis exemplifies a skin equivalent of the HPA axis, reinforcing the skin’s role in systemic stress adaptations. This is complemented by the production of vitamin D, which, alongside its receptors, impacts numerous skin functions, contributing to epidermal health and immune defense [1,2,3,4].

Moreover, the neurohormonal signaling in skin presents challenges due to its localized expression and the interdependent regulatory mechanisms of neurohormones. Hormone synthesis and receptor expression in human skin possess a unique complexity that often blurs the lines traditionally drawn in classical neuroendocrine research, emphasizing the need for a comprehensive understanding of skin neuroendocrinology and its implications in clinical dermatology. Future research should prioritize deepening our grasp of these mechanisms to inform novel therapeutic avenues for skin diseases rooted in neuroendocrine dysregulation [1,2,3,4].

## 3. Cannabinoids

### 3.1. The Endocannabinoid System

The endocannabinoid system (ECS) is an important regulatory network involved in maintaining skin homeostasis. It is composed of endogenous lipid mediators, their metabolic enzymes, and their primary receptors, cannabinoid receptor 1 (CB1) and cannabinoid receptor 2 (CB2), all of which are expressed in human skin. These components are widely distributed across the epidermis, dermis, and skin appendages, including hair follicles and sebaceous glands, supporting the existence of a functional cutaneous ECS [5,7].

Multiple cutaneous cell types, including keratinocytes, melanocytes, fibroblasts, immune cells, and sensory nerve endings, are able to both produce endocannabinoids and respond to them. This widespread expression indicates that ECS signaling operates mainly through local autocrine and paracrine mechanisms, allowing coordinated regulation of key biological processes. In particular, the ECS has been implicated in epidermal differentiation, maintenance of barrier function, melanogenesis, extracellular matrix remodeling, and hair follicle activity [5,7,23].

Beyond its physiological functions, the ECS also plays an important role in the modulation of cutaneous immune responses. Activation of cannabinoid receptors can influence cytokine production and immune cell behavior, contributing to the regulation of inflammatory processes [24]. Dysregulation of ECS signaling has been associated with several skin disorders, including atopic dermatitis, psoriasis, acne, and pruritus, highlighting its relevance in dermatological pathology [5,7,25].

Importantly, the ECS does not function in isolation but interacts with other regulatory systems present in the skin, including neuroimmune and neuroendocrine pathways. Through these interactions, it contributes to the integration of environmental stimuli and internal signals, allowing the skin to adapt to stress, inflammation, and injury.

### 3.2. The Endocannabinoid System and Wound Healing

Hemostasis, inflammation, proliferation, and remodeling/maturation are the four interrelated stages of wound healing. In the hemostasis phase, smooth muscle contraction caused by elevated calcium ions narrows blood arteries and halts bleeding, while endothelial cells produce von Willebrand factor, which promotes platelet adhesion and clot formation. Usually, this stage lasts a few minutes [18,26,27,28].

The inflammatory phase, which lasts for 0–3 days, is characterized by mast cells that use serotonin or histamine to promote vasodilation. Vimentin facilitates the migration of neutrophils and monocytes to the wound. Leukocytes and keratinocytes produce growth factors and cytokines that facilitate the phagocytosis of pathogens and damaged cells. Notably, after 12 h of insult, inflammatory cells undergo apoptosis, and pathogens are phagocytosed by neutrophils before being eliminated by macrophages. This effective elimination encourages the production of prostaglandin E2 (PGE2) and Transforming Growth Factor β1 (TGFβ1), which are essential for wound healing, and suppresses pro-inflammatory cytokines. However, the phagocytosis of apoptotic neutrophils is compromised if macrophages lack the guanine-nucleotide exchange factor Vav3. This results in decreased TGFβ1 secretion and consequent delays in myofibroblast development and wound healing [18,26,27,28].

During the three- to twelve-day proliferative phase, fibroblasts create granulation tissue and control angiogenesis and keratinocyte activity. Growth factors are also contributed by macrophages; however, vimentin shortage may interfere with fibroblast processes including collagen production and proliferation. The efferocytosis of apoptotic immune cells stimulates keratinocyte migration and reepithelialization, important for repairing the epidermal barrier [18,26,27,28]

Lastly, the remodeling/maturation phase, which lasts from three days to six months, focuses on myofibroblast-driven wound contraction and collagen replacement. Growth factors alter endothelial function by influencing mesenchymal changes via TGF-β and Notch signaling. As the extracellular matrix changes and type III collagen replaces type I collagens and elastin reappears, the phase ends with the creation of scars. Myofibroblast apoptosis facilitates closure by converting granulation tissue into a scar, although insufficient myofibroblast efferocytosis may cause fibrosis and hypertrophic scarring [18,26,27,28].

#### 3.2.1. Cell Models

Cannabis has always piqued the interest of researchers looking into its therapeutic uses, especially in relation to wound healing. The scientific studies on cannabis and its derivatives are highlighted in the recent literature reviews. Research has concentrated on how CB receptor ligands and cannabis affect keratinocyte and fibroblast cell lines. For example, Sangiovanni et al. discovered that although both substances downregulated genes overexpressed after treatment with TNF-α, *Cannabis sativa* suppressed pro-inflammatory cytokine production better than cannabidiol (CBD). In a fibroblast co-culture model, Miller et al. found increased cell migration and a 75% faster wound closure following THC and CBD exposure. Ramot et al. investigated how CB1 receptors affect keratin 6, which affects reepithelialization during wound healing. Bort et al. revealed that the CB2 agonist JWH015 lowered pro-inflammatory cytokines and expedited gap closure in scratch tests, while employing pig skin as an ex vivo model emphasized its potential therapeutic implications. According to Ruhl et al., CBD co-treatment improved hepatocyte growth factor, which is essential for keratinocyte migration, while reducing oxidative stress and preventing further differentiation in cells exposed to lipopolysaccharides [29,30,31,32,33,34,35,36,37].

Anandamide levels were shown to be lower in hypertrophic scars after surgery, which Correia-Sa et al. linked to higher inflammation and a possible tendency to scarring. They demonstrated that although its antagonist reduced collagen deposition, Arachidonyl-2′-chloroethylamide (ACEA), a CB1 agonist, increased it. This study demonstrated the intricate function of CB2 in the mechanisms involved in wound healing [38,39]. Furthermore, by lowering chronic inflammation and promoting tissue remodeling, hydrophobic flax fiber extracts containing CBD enhanced wound healing, according to Styrczewska et al. [40]. Collagen hydrogels containing silver nanoparticles and *C. sativa* extract were developed by Antezana et al., who observed that the inclusion of the cannabis extract increased biocompatibility and lowered toxicity while also reducing bacterial growth [41]. All things considered, the use of cannabis in studies shows how they may improve wound healing via a variety of biological processes [26,30,31,32].

#### 3.2.2. Animal Models

The research outlined in Section 3.2 uses a variety of animal models to examine the function of cannabinoid receptors, namely CB1 and CB2, in wound healing. Ruhl et al. examined CB1 and CB2 knockout mice and discovered that CB1 knockout animals exhibited delayed wound closure, indicating CB1’s function in regulating inflammation during healing, while CB2 knockout mice revealed elevated proinflammatory cytokines [26,30,31,32,42]. The CB2 agonist GP1a reduced collagen deposition and changed fibrogenesis, which hastened wound healing and decreased inflammatory markers, according to Zheng et al.’s research, which also showed significant increases in CB2 expression after wound damage [43]. According to Klein et al., CBD improved reepithelialization by day 7 by reducing inflammatory infiltration in rat tongue ulcers at day 3 [26,30,31,32,44]. Zhao et al. showed how a Gp1a hydrogel improved wound healing metrics and decreased inflammation by facilitating the progressive release over an 8-day period. Koyama et al. examined beta-caryophyllene’s (BCP) effects, finding better reepithelialization; however, some data revealed alterations may not be connected to CB2 activation [26,29,30,31,32,45,46]. Following mustard exposure in mice, Wohlman et al. found that pro-inflammatory proteins CB1, CB2, PPARα, and FAAH (Fatty Acid Amide Hydrolase) were upregulated, indicating that CB1 plays a role in keratinocyte proliferation [47]. Del Rio created VCE-004.8, a CB2/PPARγ agonist that affected collagen production and decreased skin fibrosis after bleomycin therapy, suggesting a particular reliance on these receptors for antifibrotic actions [48]. In vitro and in vivo, Casares et al. showed that CBD increases the antioxidant HMOX1 in keratinocytes, aiding in wound healing. Finally, McIver et al. reported no changes in healing rates across different treatments in horses, indicating potential systemic effects of CBD, although Mehrabani et al. discovered a “new formula” that greatly improved burn wound outcomes in mice. All things considered, these results demonstrate the intricate interactions between cannabinoid receptors in reducing inflammation and promoting wound healing [26,30,31,32,49].

#### 3.2.3. Human Models

A little amount of research, including observational studies and case reports, has been done on how cannabinoids, especially CBD and THC, affect human wound healing. A patient taking oral CBD-dominant and THC-dominant oils for a persistent pressure ulcer showed remarkable improvement after two weeks and virtually full closure by two months, according to a noteworthy case report by Diaz et al. Nevertheless, using a foam-padded dressing might skew the results [26,29,30,31,32,50]. Maida et al. also looked at topical cannabis-based medications (TCBMs) for the treatment of nonuremic calciphylaxis ulcers and venous stasis ulcers. In their trial on venous ulcers, 14 patients with 16 ulcers were treated with TCBM in addition to compression bandaging, and 11 of them had full wound closure after a median of 34 days [51]. Two patients in a different research on nonuremic calciphylaxis had their ulcers completely closed in an average of 76.3 days. The lack of control groups in these studies, however, makes it more difficult to attribute healing to TCBMs [26,30,31,32]. Three patients in a case study on epidermolysis bullosa (EB) reported considerable pain alleviation and decreased opioid usage after applying different topical CBD formulations; however, the results are limited by the absence of objective data and standardization. In an international cross-sectional research, Schräder et al. found that both topical and oral CBD enhanced wound healing and reduced pain and pruritus in 71 EB patients and their caregivers [26,29,30,31,32,52]. Finally, Palmieri et al. assessed the effects of CBD ointment in 20 patients with atopic dermatitis and moderate to severe psoriasis. Improvements in skin hydration and suppleness and a decrease in scars and imperfections were seen after three months of therapy, coupled with a drop in psoriasis severity ratings [53]. Overall, even if early research and anecdotal data point to possible advantages of cannabis in wound healing, further research is required due to the absence of strict scientific controls and standardized treatment procedures [26,30,31,32].

### 3.3. Therapeutic Applications in Barrier Defect Diseases

Skin barrier disorders, such as atopic dermatitis, psoriasis, and ichthyosis, involve epidermal dysfunction and chronic inflammation, for which there are still no fully effective long-term therapies. Therefore, the search for new therapeutic strategies is crucial. Recently, dysregulation of the cutaneous ECS has been shown to contribute to several highly prevalent diseases and disorders, including atopic dermatitis [54], psoriasis [38], scleroderma, acne [55], hair growth disorders [56] and pigmentation disorders [57], keratin diseases [58], various tumors [25], and pruritus [59]. Consequently, the ECS has become a promising therapeutic target for skin conditions in which the skin barrier is compromised.

THC, CBD, and cannabigerol (CBG) are the main phytocannabinoids of the *Cannabis sativa* plant that interact with the human ECS with distinct effects. CBD and THC act on CB1 and CB2 receptors in keratinocytes, which are involved in lipid synthesis [58], regulating proliferation and differentiation, thus contributing to the restoration of epidermal homeostasis [7,60]. Furthermore, ECS signaling influences key molecular pathways involved in epidermal differentiation and stratum corneum integrity [56,61]. More recent evidence indicates that phytocannabinoids can enhance the expression of proteins related to the skin barrier and promote lipid production, thereby improving the integrity of the stratum corneum [5,7,56,62].

From a therapeutic perspective, cannabinoids exert potent anti-inflammatory and immunomodulatory actions of great relevance in skin conditions such as atopic dermatitis and psoriasis [63], where barrier disruption, inflammation, and pruritus coexist. Preclinical studies have demonstrated that CBD and THC reduce the production of pro-inflammatory cytokines and chemokines in keratinocytes, including Th2- and Th17-mediated responses, leading to the attenuation of both acute and chronic inflammation [38,64]. Furthermore, several studies have revealed that cannabigerol improved dermatitis severity, epidermal thickness, and mast cell count by reducing inflammatory cytokines (Tslp, IL1b, IL4, IL6, IL13, IL17, IL18, IL22, and IL33) and the signaling factors p-IκBα, NF-κB, and p-NF-κB [65]. Similarly, the therapeutic effect of CBD appears to be associated with the suppression of key inflammatory regulators in the MAPK ERK, JNK, and p38 signaling pathways [45,66]. Additionally, in atopic dermatitis, topical formulations containing CBD, alone or in combination with other cannabinoids such as cannabigerol, have been associated with improvements in skin hydration and elasticity, as well as reductions in transepidermal water loss and pruritus [67]. Recent clinical evidence further supports these findings. A prospective study evaluating a cannabidiol-based topical formulation in atopic-prone skin demonstrated significant reductions in disease severity, pruritus, and skin dryness, together with good tolerability, highlighting the translational potential of cannabinoid-based therapies in dermatology [68]. BCP has also been described as suppressing the NF-κB/MAPK and IL-4/TSLP pathways, enhancing Nrf2-mediated antioxidant defenses, and accelerating reepithelialization and collagen remodeling in chronic inflammatory skin disorders, including atopic dermatitis, psoriasis, and acne [54]. In this regard, in psoriasis, cannabinoids have also been shown to inhibit the hyperproliferation of keratinocytes and normalize epidermal renewal, suggesting possible disease-modifying properties rather than purely symptomatic relief [7,38,69].

Regarding their antinociceptive potential, an abundant distribution of cannabinoid receptors has been described in cutaneous nerve fibers and mast cells, suggesting an anti-inflammatory and antinociceptive action of cannabinoid receptor agonists and indicating their therapeutic potential [38,61,70]. It is worth noting that the components of the ECS are expressed in cutaneous sensory nerves, and their activation has been shown to reduce itch signaling through both peripheral and central mechanisms [38,60,71]. In vitro studies have revealed significant overexpression of cannabinoid-metabolizing enzymes, such as FAAH and monoacylglycerol lipase (MAGL), in psoriatic lesions, and pharmacological inhibition of these enzymes can significantly reduce psoriasiform inflammation and pruritus [72]. In this context, cannabinoid-based therapies may offer a dual benefit by relieving itching while simultaneously promoting skin barrier repair. Therefore, these effects could allow cannabinoids to act not only as symptomatic treatments but also as modulators of the disease’s pathophysiology.

On the other hand, in xerotic and ichthyotic conditions, observational studies using cannabinoid-based formulations described a reduction in itching and an improvement in eczema [73]. Other studies on this type of skin alteration have shown that the use of cannabinoids can increase epidermal lipid synthesis and reduce transepidermal water loss and the appearance of redness, which would support their use as adjunctive therapies in chronic deterioration of the skin barrier [74,75,76].

Additionally, studies have been conducted to analyze the antimicrobial activity of CBD and CBG, including their ability to inhibit biofilm formation and promote its elimination. Recent work has reported that both molecules exhibit activity against planktonic bacteria and biofilms, as well as their ability to inhibit staphylococcal adhesion to keratinocytes, without negative effects on the skin microbiota [77].

Despite these promising findings, limitations currently restrict their clinical application. Most available data come from in vitro studies, animal models, or small-scale clinical trials, highlighting the need for large-scale randomized controlled trials. Furthermore, the variability in the composition, dosage, and delivery systems of cannabinoids hinders standardization and comparison between studies. Challenges related to skin penetration, stability, and regulatory frameworks further limit their widespread adoption [7,56,60]. In conclusion, modulation of the ECS represents a novel and promising strategy for the treatment of skin barrier disorders. While accumulating evidence supports the beneficial effects of cannabinoids on epidermal structure, inflammation, and pruritus, further research is needed to validate these findings and optimize their clinical application.

### 3.4. Cosmeceuticals and Novel Formulations

The increasing interest in cannabinoids in dermatology has promoted their incorporation into cosmeceutical products. The main challenge lies in the intrinsic physicochemical properties of cannabinoids, their high lipophilicity, poor water solubility, and susceptibility to oxidative degradation. These properties significantly restrict their cutaneous penetration to the stratum corneum and bioavailability in deeper layers of the skin [74]. These limitations are not limited to CBD, but also to other phytocannabinoids such as CBG, cannabichromene (CBC), and THC.

CBD has emerged as a promising bioactive compound in skincare formulations due to its multifaceted effects on skin physiology, including antioxidant, anti-inflammatory, and sebostatic properties [74]. Novel investigations aimed to treat aging, acne, and inflammatory skin conditions with CBD.

Recent developments in formulations have focused on enhancing the stability, bioavailability, and skin penetration of cannabinoids. Given their lipophilic nature, cannabinoids are designed for incorporation into lipid-based delivery systems, as creams, ointments, and nanoemulsions [75].

Nevertheless, the stratum corneum represents the principal barrier to transdermal delivery of cannabinoids in lipid-based formulations. Cannabinoids tend to accumulate within the lipid matrix of the stratum corneum, leading to limited diffusion into deeper epidermal and dermal layers. This results in suboptimal therapeutic concentrations at target sites [78]. Additionally, THC exhibits greater chemical instability compared to CBD, suffering progressive degradation in conventional bases such as creams, compromising formulation efficacy [79].

To overcome these challenges, advanced delivery systems based on nanotechnology have emerged as a promising strategy. Nanostructured lipid carriers (NLCs), typically ranging from 160 to 200 nm, enable near-complete encapsulation of cannabinoids, improving their photostability and enhancing anti-inflammatory activity while maintaining cellular viability [80]. Similarly, deformable liposomes and hybrid systems such as drug-in-micelles-in-liposomes (DiMiLs) have demonstrated superior epidermal penetration compared to conventional liposomal formulations, allowing to traverse intercellular lipid domains [81].

Microemulgel systems represent another effective approach, combining the solubilisation capacity of microemulsions with the favorable rheological properties of hydrogels. These formulations, with droplet sizes in the nanometric range (~35 nm), allow controlled and sustained cannabinoid release, achieving measurable transdermal penetration over extended periods [82]. In parallel, emerging platforms such as Pickering emulsions, nanomicelles, and cryogels have shown high levels of evidence for improving penetration, stability, and safety profiles of topical cannabinoids [83]. Hydrogels enriched with *Cannabis sativa* extracts have also demonstrated favorable chemical properties, as well as beneficial effects on skin cell viability, suggesting their potential as innovative delivery platforms [75]. Nevertheless, conventional semisolid formulations may not adequately preserve cannabinoid stability.

Beyond formulation strategies, the selection of the cannabinoid may influence both efficacy and delivery. Minor cannabinoids exhibit distinct biological and physicochemical behaviors that may be exploited for targeted cosmeceutical applications. In contrast, CBG needs advanced delivery systems to improve solubility and penetration [84]. THC, while biologically active, is limited in cosmeceuticals by regulatory restrictions and by a higher degradation rate and variability in topical absorption.

Pharmacokinetic data from commercial topical formulations further illustrate these challenges. Transdermal absorption of cannabinoids has been demonstrated, but it is dependent on the formulation vehicle, excipient composition, and the presence of penetration enhancers. Preparations incorporating lipophilic vehicles and agents such as vitamin E have shown improved systemic exposure, whereas other formulations yield minimal or inconsistent absorption [85]. Notably, despite detectable systemic levels of CBD, topical application does not appear to produce psychotropic effects, as THC concentrations remain below quantifiable thresholds in most cases.

Despite these advances, several critical limitations persist. The lack of standardization in formulation, variability in penetration in the skin, and small clinical data continue to limit the validation of these products [86,87]. Furthermore, regulatory frameworks remain fragmented, with no topical cannabinoid formulations currently approved by major agencies such as the FDA for dermatological indications [88].

**Figure 6:**
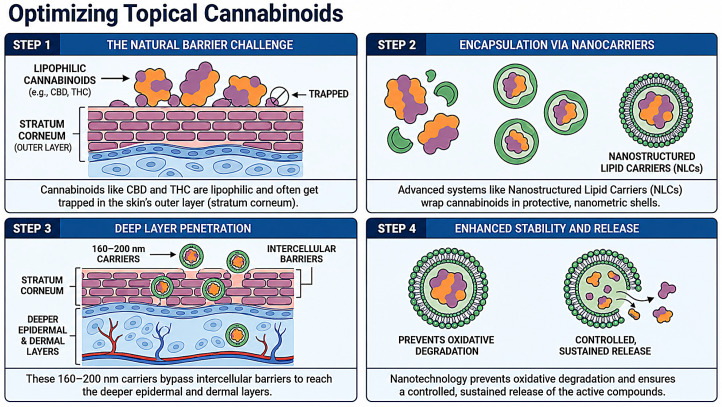
Strategies for enhancing the penetration of cannabinoids in the skin.

Future research should focus on optimizing formulation strategies to enhance dermal penetration while preserving compound stability, particularly through the integration of nanocarriers and hybrid delivery systems. In addition, the exploration of minor cannabinoids such as CBG and CBC represents a promising strategy for the development of targeted cosmeceuticals with differentiated mechanisms of action. Overcoming current pharmacotechnical barriers will be essential to foster the potential of topical cannabinoids in dermatology (Figure 6).

## 4. Psychedelics

Serotonergic psychedelics, such as psilocybin, mescaline, N,N-dimethyltryptamine (DMT) and lysergic acid diethylamide (LSD), function primarily as agonists of the 5-HTR2A receptor, inducing alterations in perception and states of consciousness. While these substances have historical roots in indigenous rituals and recreational use, scientific and clinical interest has surged over the past decade due to their therapeutic potential for psychiatric conditions, including treatment-resistant depression, anxiety, and substance use disorders [89]. 5-HTR receptors are most widely expressed in the mammalian body localized in nearly every tissue, including epithelial and immune cells; they play a critical role in maintaining cellular homeostasis and regulating inflammatory responses. Consequently, investigating the physiological interactions between psychedelic compounds and the dermis represents an interesting approach for dermatological research [90].

### 4.1. The Serotonergic System and 5HT Receptors

5-Hydroxytryptamine, most known by the name of serotonin or 5-HT, is distributed widely throughout the body and has been related to the regulation of different physiological states and behaviors [91]. Moreover, this molecule seems to play an important role in skin physiology; it has been reported to have an influence on multiple processes such as migration, maturation, mitosis and inflammatory responses [92].

The receptors of serotonin (5HTR) are located on the surface of the cell membrane and are divided into seven families. Coupled to G-proteins, these receptors can influence the activity of adenylate cyclases (5-HTR7, 5-HTR1) and PI-hydrolases (5-HTR2A), act as ion channels (5-HTR3) and regulate the levels of c-AMP (5HTR1A, 5HTR7) [93,94].

Some psychedelic drugs like psilocybin or mescaline target these 5-HT receptors, specifically the 5-HTR2A, a low-affinity serotonin receptor that is only activated by high levels of 5-HT, which enhance the activity of PI-hydrolases and increase the levels of intracellular Ca++, stimulating kinase activity, cell division and apoptosis [93,95,96].

5-HT receptors are expressed throughout both the central and peripheral nervous system; indeed, they have been demonstrated to be expressed in skin cells such as fibroblasts, melanocytes and keratinocytes [90]. The serotonergic system in skin could be involved in the pathology and biology of the dermis; the 5-HT, which is mainly synthesized by epidermal melanocytes, Merkel cells and mast cells, may influence the differentiation and lifespan of different types of skin cells via 5HT receptors, also playing a leading role in immune response and inflammation regulation [92].

Furthermore, the expression of certain 5-HT receptor subtypes may change depending on the cellular physiological and pathological state. For instance, Park et al. (2021) characterized 5-HTR receptors in mouse skin samples and found that, in healthy skin samples, 5-HTR2A, 5-HTR2B and 5-HTR7 were barely expressed whereas their expressions were significantly increased in the dermatitis-induced model (MC903) [97]. Moreover, while other studies reported the expression of 5-HT2R2A, 5-HTR1A, 5-HTR1B and 5-HTR7 in epidermal keratinocytes and dermal fibroblasts, in the immortalized HaCat keratinocyte cell line no expression was detected [90]. Consequently, future research should prioritize the characterization of 5-HT receptors in different types of skin cells, considering distinct states of differentiation, pathologies and age.

Research into applications of 5-HT agonist drugs could be an interesting approach for treatments of different skin pathologies. Furthermore, compounds that cannot cross into the bloodstream could gain significant relevance as the side effects would be less severe [92].

### 4.2. Anti-Aging Properties

The cutaneous aging is a particularly relevant process; the skin is not only a protective barrier against external exposures but also is a mirror of physiological and molecular changes. The cellular aging is manifested by senescence, telomere shortening, oxidate stress, chronic inflammation and alterations of the DNA repair mechanism, leading to the deterioration and atrophy of the epidermis and the loss of the protective function [98,99,100].

As the serotonergic system and 5HT receptors seem to be important in the development of cellular aging, there is growing interest in psychedelic compounds, particularly those acting as agonists of the serotonin 5-HT2A receptor due to their potential as modulators of these processes [11,92,101].

Psilocybin is an indole-based secondary metabolite produced by more than 200 species of mushrooms. Pharmacologically, psilocybin acts as a prodrug that is transformed into psilocin, which serves as an agonist for serotonin 5-HT receptors. Modern medical interest in this compound has surged due to clinical studies indicating its significant therapeutic potential for treating central nervous system disorders such as depression, anxiety, and addictions [102].

Two recent pieces of research studied the potential anti-aging properties of psilocybin and psilocin in fibroblasts and mice. Norouzkhani et al. [12] performed in vitro assays with BJ5Ta primary human fibroblasts under an induced high-glucose/high-lipid (HGHL) diet aging model; the results reported cell reactions to different psilocybin doses, improving fibroblast’s health by enhancing cell viability, promoting regenerative processes, and reducing apoptosis, senescence and inflammatory markers such as IL-1β, IL-6, and COX [12]. Moreover, psilocybin was able to restore the cell cycle dynamics as HGHL provoked S phase arrest; additionally, it seems to induce the expression of elastin, an important ECM component responsible for maintaining skin structure and elasticity; however, it showed no significant improvements in fibroblast’s migration and wound healing [12].

Furthermore, Kato et al. [11] studied the impact of psilocin in human fetal lung and adult skin fibroblasts using a validated model of replicative senescence [11,103]. The results showed an increase in cell lifespan more than 50% compared to the vehicle in both cell lines. Nevertheless, induction of senescence when reaching proliferative potential exhaustion occurred in both control and treatment groups; however, senescence seems to be delayed in psilocin-treated cells with no evidence of oncogenic transformation, and these results were consistent with the reduction in βgal activity and cell cycle arrest markers (p21, p16) along with the increase in proliferation (PCNA) and DNA replication markers (pRB). High levels of SIRT1, which plays a lead role in aging, metabolism and stress response, and Nrf2 (regulator of oxidant responses) were detected in psilocin treatment; likewise, assays showed a reduction in GADD45a (DNA damage marker) and Nox4 (oxidant production). Lastly, an evaluation of the telomere length was carried out, exhibiting the preservation of telomere length in the psilocin-treated cells [11].

Psilocin seems to have an impact on in vitro assays; data suggest the triggering of signaling pathways associated with cellular aging, increasing lifespan and delaying senescence. In order to evaluate these effects in vivo, Kato et al. [11] completed their research using 19-month-old mice (equivalent to 60/65 human years) to evaluate the impact of psilocybin in a more relevant model. Psilocybin-treated mice showed a higher rate survival (80%) compared to the vehicle (30%), in addition to the exhibition of improvements in the overall aspect of mice, such as fur quality, hair growth and less appearance of white hair [11].

In summary, research indicates that psilocin and psilocybin may have an impact on cell aging processes, suggesting a possible new therapeutic use for these compounds. However, there is no evidence that these effects would work in a real environment like human skin. Consequently, more research in this field should be carried out with better representative experimental models and more detailed in vivo assays in animal models.

### 4.3. Anti-Inflammatory

Inflammation is an endogenous defense and repair mechanism triggered by physical, chemical, or biological insults to neutralize offensive agents and facilitate tissue healing [104,105]. In this process are involved both innate and adaptive immune responses; the innate immune response acts within minutes to hours after stimulus, recruiting immune cells and promoting inflammation via cytokine release and recruitment of immune cell such as macrophages or dendritic cells [105,106]. In pathological conditions, immune responses could become uncontrolled, leading to tissue damage; for that reason, traditional treatments aim to reduce inflammation or suppress immune activity [107].

It is well known that the serotonergic system is involved in inflammation response, and it has been related with gut, liver and pancreas damage [108]. Serotonin levels in blood seem to have a correlation with expression of proinflammatory cytokines IL-6 and TNF-α, also having a relation with modulation of cytokine and chemokine production in monocytes via 5-HT3, 5-HT4, and 5-HT7 receptors [109,110].

Nevertheless, (R)-DOI, a potent 5-HTR2A agonist related to mescaline with high hallucinogenic effects, demonstrated extreme inhibition of TNF-α on rat smooth muscle cells. The agonist not only repressed TNF-α but also blocked activation of NF-κB and nitric oxide synthase; (R)-DOI also induced the expression of intracellular adhesion molecule-1 (ICAM-1), vascular cell adhesion molecule-1 (VCAM-1), and inflammatory cytokine IL-6 [111]. Additionally, in vivo studies in mice with (R)-DOI treatment corroborated the results obtained from in vitro [112].

Flanagan and Nichols [108] suggested an hypothesis for the difference between serotonin pro-inflammatory responses and the (R)-DOI anti-inflammatory properties through the same receptors: some compounds may activate alternative pathways of a receptor by inducing different conformations, and this is called functional selectivity [108,113].

Other psychedelic drugs like psylocibin and psilocin have proven anti-inflammatory effects; psilocin was tested in lipopolysaccharide (LPS)-activated macrophages resulting in a decrease in TNF-α and an increase in IL-10, as well in aged fibroblasts where a reduction in IL-6, IL-1β, COX-2 and TNF-α was reported [12,114].

TNF-α inflammatory pathways are implicated in some common skin pathologies like psoriasis, and compounds that block these vias are being studied as treatments for diseases [115].

### 4.4. Photoprotection and Wound Healing

The skin is the largest organ of the body and the most exposed one to the sunlight; the excess of UV radiation is responsible of sunburns, inflammation, photoimmunosuppression, aging and skin cancer. The most established way to prevent UV damage in skin involves using protectors like sunscreen; however, it is interesting to study the regulatory mechanisms of skin against the UV damage to develop new paths for further therapies [116].

Photocarcinogenesis is the most common cancer in humans and has kept increasing in the last few decades, with UV radiation having the main responsibility among other factors like viruses, inflammation or genetics, due to the irreversible genetic alterations and immunosuppressive character of UV radiation [117,118]. 5-HTR receptors participate in the skin reaction to UVR; for instance, 5HTR1 is known to be involved in T and B cell proliferation and the expression of cytokines is stimulated via 5-HTR3, 5-HTR4 and 5-HTR7 receptors [119]. Moreover, cis-UCA (cis-urocanic acid) acts as a competitive agonist of 5-HT2A receptor and is described as playing an important role in UV-induced immunosuppression. However, some findings indicate that cis-UCA and 5-HT immune modulation properties are via different pathways [120,121,122].

Some research has been carried out using 5HTR agonists such as zolmitriptan, a non-reported psychedelic agonist of 5HTR1B that blocked immunosuppression in mice treated with cis-UCA. Another compound, 1-NPZ (1-naphthylpiperazine), agonist of 5-HTR2A and 5-HTR1A receptors with potential psychedelic effects, also prevented the immunosuppression provoked by cis-UCA [119].

Sreevidya et al. [118] studied the synergistic action of 1-NPZ and PAF (platelet-activating factor) in mice, showing prevention of UVR skin lesions [118]. In addition, other research suggested that 1-NPZ and PAF modulate DNA repair by repairing CPDs (Cyclobutene Pyrimidine Dimers) and 6-4 photoproduct and inhibiting ROS production [118,120].

As the 5-HTR agonist has proven anti-photocarcinogenesis properties, it would be interesting that further research would encourage evaluating the possible effects of natural less-toxic psychedelic compounds targeting 5-HTR2A receptors such as psilocybin, mescaline or DMT.

Ayahuasca is an ancient beverage extracted from *Banisteriopsis caapi* (*B. caapi*) and leaves of *Psychotria viridis* (*P. viridis*), and it is used as a recreative drug due to their its hallucinogenic effects [123,124]. DMT is the main psychoactive compound of “Ayahuasca”, acting as an agonist of 5-HTR2A and SIGMAR1 receptors [125].

There is a special interest in researching natural compounds that improve wound healing; Gonçalves et al. [123] decided to test ayahuasca properties in wound-healing assays supported by the scientific background, as evidence has been described about the anti-inflammatory, antioxidant and anti-microbial potential of DMT and other compounds of ayahuasca beverages [123,126,127]. Results showed that the different combinations of *M. hostilis*, *P. viridis* and *B. caapi* enhance fibroblast’s migration; however, we cannot assure that these effects are only due to DMT, but they also could be provoked by other natural extracts present in the plants [123].

It is worth pointing out that 5-HT acts as a dermal growth and proliferation factor in fibroblasts and keratinocytes; nevertheless, in immortalized epidermal melanocytes it induces growth in the absence of growth supplements and inhibits proliferation in the presence of these factors. These effects could be due to accumulation of cAMP via 5-HTR receptors on the antagonism of 5-HT and growth factors [90,118].

### 4.5. Applications and Drawbacks

Recent scientific evidence suggests a paradigm shift in the therapeutic applications of psychedelic drugs. Results indicate potential benefits in peripheral tissues, such as the skin, opening doors to new pharmacological applications. However, the development of efficient dermatological formulations faces several technical challenges, including the instability of these compounds, the risk of systemic absorption (reaching the bloodstream), and extremely strict regulations regarding psychedelic substances.

The design of a pharmacological formulation is essential not only to preserve and enhance a compound’s properties but also to control its delivery and stability. Most psychedelic drugs have a molecular weight under 500 Da (the 500 Da rule) and a lipophilic profile; these characteristics enable them to penetrate the stratum corneum of the skin [128,129]. However, the chemical stability of certain compounds, such as psilocin, is very low, which is a primary consideration during formulation [130]. In this context, research must focus on systems that maintain the stability and properties of the compound, allowing the drug to permeate the epidermis without entering the bloodstream.

Nanoparticles represent one of the most promising technologies for the topical delivery of alkaloids, including solid lipid nanoparticles (SLNs), liposomes, and NLCs. These nano-formulations improve skin occlusion and hydration while protecting the compound from external stressors like UV radiation or oxygen, thereby increasing stability [129,131]. Additionally, other options include using hydrogel matrices or nanoemulgels, particularly interesting when using polymer-based hydrogels such as chitosan, alginate, or hyaluronic acid, which may provide synergistic effects with psychedelic drugs. Moreover, NLCs are capable of concentrating the active principle in the upper layers of the skin, minimizing systemic permeability [129,132].

Regarding safety profiles, hallucinogenic effects would likely be irrelevant due to enhanced localized topical delivery combined with low drug dosages. Although technical data sheets from Cayman Chemical indicate that compounds like psilocin or psilocybin cause skin irritation in their pure state, further research is required to study the potential dermatological side effects of these substances in standard topical doses.

In conclusion, the prospect of utilizing psychedelic drugs in dermatology remains far from reality. Research into the potential modulatory effects of these compounds is still recent, and there is currently no evidence demonstrating their efficacy on actual human skin. Furthermore, stringent regulatory restrictions pose a significant hurdle for bringing any such pharmacological formulation to market. However, while challenging, it is not impossible; for example, Colognesi et al. [133] recently completed a Phase 1 study evaluating low, non-psychedelic micro-doses (0.5–2 mg) of psilocybin [133]. Nevertheless, even if these specific drugs never reach the market, the study of the serotonergic system, the relation with skin aging and how compounds might modulate it is essential for opening new pathways toward the discovery of novel treatments. In addition, future research should focus on formulation strategies that minimize systemic absorption, such as the use of nanocarriers or controlled-release delivery systems, in order to limit potential central nervous system effects associated with serotonergic activation.

## 5. Antidepressants

Antidepressants have long been used in dermatology, particularly in psychodermatology, due to their efficacy in treating psychiatric disorders frequently associated with skin disease, including depression, anxiety, obsessive-compulsive symptoms, body dysmorphic disorder, and psychogenic excoriations. However, their role is not limited to these contexts. Antidepressants comprise several pharmacological classes, including selective serotonin reuptake inhibitors (SSRIs) such as fluoxetine, sertraline, and paroxetine, as well as tricyclic antidepressants (TCAs) such as doxepin, amitriptyline, imipramine, and desipramine. Other agents with atypical or mixed mechanisms, including clomipramine, may also exert relevant effects in dermatological contexts. Several antidepressants, particularly SSRIs and some TCAs, show antipruritic, immunomodulatory, and, in some experimental contexts, pro-reparative effects on the skin.

This means antidepressants are being considered not only as supportive agents for psychodermatologic burden, but also as potentially relevant drugs for cutaneous inflammation, epidermal signaling, and tissue repair [8,134].

That said, the strength of the evidence is uneven. Experimental studies describe effects on keratinocytes, inflammatory mediators, and serotonin-related pathways in the skin, but clinical support is much stronger in areas such as chronic pruritus and psychodermatologic disorders. Evidence-mapping studies also highlight the limited number of well-designed randomized trials in this field, which makes it difficult to draw firm clinical conclusions at this stage [9,10]. Importantly, the observed effects may differ depending on whether antidepressants are administrated systemically or applied topically.

### 5.1. Antidepressants and Cutaneous Molecular Mechanisms

Only a limited number of studies have explored direct effects of antidepressants on skin cells, and most come from in vitro or ex vivo models. In an inflamed HaCaT keratinocyte model, Curzytek [135] found that inflammatory stimuli increased IL-1β, IL-6, CCL2, CXCL8, and ICAM-1, while reducing E-cadherin expression. In the same study, imipramine, desipramine, and fluoxetine reduced IL-1β, CCL2, and ICAM-1, and fluoxetine restored the decline in E-cadherin caused by LPS. Therefore, some antidepressants may modulate inflammatory and adhesion-related responses in keratinocytes, although direct effects on skin barrier function were not demonstrated [8,9,135].

Serotonin signaling appears to be especially relevant. Changes in local serotonergic signaling, such as elevated serotonin-positive platelets and elevated expression of serotonin transporter (SERT) and 5-HT2A receptor-positive cells in inflammatory skin, have been linked to human allergic contact dermatitis. These observations provide biological support for the hypothesis that drugs targeting serotonin pathways may influence cutaneous inflammation and epidermal responses [136].

Further support comes from transcriptomic and phosphoproteomic studies. Rodriguez-Barucg et al. [8] showed that fluoxetine accelerated wound closure and promoted skin cell proliferation, alongside changes in gene expression and protein phosphorylation related to serotonin signaling, cell cycle regulation, and tissue morphogenesis. Rather than acting through a single pathway, SSRIs seem to produce broader shifts in cellular signaling networks [8].

At the same time, not all cutaneous effects of antidepressants appear potentially beneficial. Wolf et al. [13] showed that several cationic amphiphilic drugs, such as clomipramine, paroxetine, and desipramine, activated MRGPRX2 and induced mast cell degranulation in experimental systems. Intradermal exposure also produced scratching behavior in mice and wheal-and-flare reactions in humans. These results support a possible mechanism by which some antidepressants may contribute to non-IgE-mediated cutaneous reactions in specific settings, implying that their cutaneous effects are probably context-dependent and drug-specific rather than uniformly beneficial [13,14].

### 5.2. Immune Regulation and Anti-Inflammatory Effects

The clearest evidence for anti-inflammatory activity comes from contact hypersensitivity (CHS) models, which represent a form of type IV delayed hypersensitivity reaction, where both fluoxetine and desipramine have demonstrated consistent immunomodulatory effects in epidermal cell types and in vivo studies following systemic administration. According to Kubera et al. [137], these drugs significantly suppressed contact sensitivity reactions in mice delivered via intraperitoneal injection, with associated decreases in lymphoid organ reactivity and increases in IL-10 production, suggesting that antidepressants may downregulate T cell-mediated immune responses while favoring anti-inflammatory cytokine profiles [137].

Similarly, Curzytek et al. [138] strengthened this line of research by demonstrating that antidepressants lowered splenocyte proliferation, raised IL-10, decreased IL-6 and IFN-γ production, and prevented 2,4-dinitrofluorobenzene-induced CHS. Since several inflammatory dermatoses involve similar cytokine and T-cell axes, the authors’ conclusion that antidepressants inhibit Tc1-dependent immune responses may be relevant beyond the CHS model. Rather than acting only through the central nervous system, these drugs appear capable of modifying peripheral immune responses as well [138].

A later study by Curzytek et al. [139] offered additional molecular insight by demonstrating that animals lacking CD8+T cells or NKT cells lost the immunosuppressive effect of antidepressants on CHS, while γδ T cells did not seem to be necessary for this response. This points to a selective effect on particular immune populations involved in cutaneous inflammation, instead of a broad and nonspecific immunosuppressive action [139].

At the epidermal level, the HaCaT study by Curzytek et al. [135] demonstrated that antidepressants decreased keratinocyte synthesis of CCL2 and IL-1β and lowered ICAM-1 expression. These findings provide a viable biological foundation for the anti-inflammatory effects seen in animal models since keratinocytes are crucial in determining skin immune responses [135]. Additionally, more recent translational research has indicated potential significance in inflammation similar to psoriasis. Likewise, Fluvoxamine was found to be a potential option when Martins et al. [140] evaluated topical SSRIs in keratinocytes, monocytes, and an imiquimod-induced psoriasiform model [140]. Although these findings are still preclinical, they expand the potential dermatologic relevance of antidepressants beyond itch and psychodermatologic indications alone.

Current evidence therefore supports an anti-inflammatory role for some antidepressants, particularly SSRIs and certain TCAs. What remains unclear is whether these effects, demonstrated consistently in mice and cell cultures, translate to meaningful clinical benefit in conditions like psoriasis or atopic dermatitis.

### 5.3. Wound Healing and Cell Proliferation

Wound healing is one of the most interesting emerging areas in antidepressant repurposing. So far, fluoxetine has attracted the most significant attention. Nguyen et al. [141] demonstrated that topical fluoxetine improved cutaneous wound healing, increased angiogenesis, enhanced keratinocyte migration, and changed the wound environment to a less inflammatory state in diabetic mouse models. The drug appeared to influence several phases of repair like reepithelialization, inflammation, and tissue remodeling [141].

Its potential benefits have also been investigated in infected wounds. Topical fluoxetine may function as a non-antibiotic supplementary therapy by combining direct antimicrobial activity with effects on wound behavior, according to Yoon et al. [142]. This supports the idea that fluoxetine may have multifunctional topical activities relevant to chronic or complicated wounds, but it does not place the drug among standard wound-healing treatments [142].

More recently, Rodriguez-Barucg et al. [8] found that in experimental systems, fluoxetine sped up wound closure and promoted skin cell proliferation. These effects were accompanied by changes in serotonin signaling, gene expression, and phosphorylation pathways involved in tissue repair. Currently, fluoxetine is the antidepressant with the most advanced preclinical evidence in this field [8].

However, there is a complex association between serotonergic signaling and wound healing. According to Sadiq et al. [143], endogenous serotonin contributes to healing after thermal injury and disrupting this pathway can modify reepithelialization. Therefore, several variables, including receptor subtype, exposure timing, drug concentration, wound type, and inflammatory setting, may influence the final effect of an antidepressant. So, while fluoxetine and related agents show promise, the evidence remains preclinical and should be interpreted with caution before being applied to routine wound care [143].

Fluoxetine is clearly the standout candidate here, but it is still an open question if other SSRIs share these properties or whether any of them are effective in treating chronic wounds in humans.

### 5.4. Clinical Efficacy and Safety Profile

From a clinical perspective, one of the strongest dermatologic uses of antidepressants is treating chronic pruritus. A review by Kouwenhoven et al. [10] indicates that oral antidepressants may be considered in patients whose itching does not improve with topical therapy or antihistamines, especially in cases of uremic, cholestatic and paraneoplastic pruritus. On the other hand, the authors also pointed out important limitations in the evidence base, including inconsistent findings, limited comparative proof, and lack of long-term randomized controlled trials [10].

Sertraline is one of the SSRIs which has shown particularly relevant effects in cholestatic pruritus. Mayo et al. [144] reported that sertraline was effective and well tolerated in patients with chronic liver disease, which fits with the idea that serotonergic pathways play a role in itch perception. Paroxetine has also shown benefit. In a randomized controlled trial, Zylicz et al. [145] found that it reduced severe non-dermatological pruritus with a relatively rapid onset of action. Both studies support the prescription of specific SSRIs as systemic antipruritic medicines, particularly when conventional options fail or when psychiatric comorbidity is also present [144,145].

Topical doxepin remains one of the best-known antidepressant-based therapies in dermatology. Early trials in atopic dermatitis showed significant reductions in pruritus together with an acceptable short-term safety profile. Other studies also reported benefit in eczematous dermatitis more broadly, although drowsiness and local stinging were common adverse effects. Clinical benefit, however, does not seem universal across all forms of itch. For example, doxepin cream did not reduce itch in burn-scar patients, suggesting that its usefulness depends heavily on the underlying disease and mechanism of pruritus [146,147].

Outside the setting of itch, the clinical evidence becomes much weaker. Reviews on psychopharmacology in dermatology continue to support antidepressants mainly for psychodermatologic disorders and symptom control, rather than as primary disease-modifying therapies for inflammatory skin disease. Recent reviews have mentioned potential benefits in conditions such as psoriasis or hidradenitis suppurativa, but these claims rely much more on mechanistic and observational data than on robust clinical trials. Evidence-mapping studies likewise show that randomized controlled trials in primary psychodermatologic disorders are still relatively scarce [148,149].

Safety also needs to be considered carefully. SSRIs are generally better tolerated than TCAs from a systemic point of view, but cutaneous adverse effects still occur. Krasowska et al. [150] reviewed skin reactions associated with SSRIs and described manifestations ranging from pruritus, urticaria, and photosensitivity to severe events such as erythema multiforme, vasculitis, Stevens–Johnson syndrome, and toxic epidermal necrolysis. Stevens–Johnson syndrome (SJS) and toxic epidermal necrolysis (TEN) represent the most severe forms of cutaneous adverse drug reactions. These conditions are rare but potentially life-threatening, typically characterized by widespread epidermal necrosis, blistering, and mucosal involvement, often preceded by flu-like symptoms. They are most commonly drug-induced and require immediate recognition, withdrawal of the offending agent, and supportive care, frequently in specialized settings [151,152].

These reactions may involve different immunological mechanisms, including type I–IV hypersensitivity responses, ranging from immediate IgE-mediated reactions to delayed T cell-mediated responses, depending on the drug and clinical context [150,153,154].

In addition, interindividual variability, including genetic predisposition, may influence susceptibility to these reactions, as pharmacogenetic factors such as specific HLA alleles have been associated with an increased risk of severe cutaneous adverse reactions [155,156,157].

A broader review of SSRI adverse effects similarly emphasized that, although uncommon, serious skin reactions may be life-threatening and require early recognition. In addition, the MRGPRX2 findings offer a biologically plausible explanation for certain immediate non-IgE-mediated skin reactions induced by some antidepressants [150,158].

Recent evidence has also explored potential long-term dermatological safety concerns associated with antidepressants. A systematic review and meta-analysis found no significant association between antidepressant use and skin cancer risk, although the authors highlighted the need for further high-quality studies considering potential confounding factors [159].

In clinical practice, antidepressants are therefore best supported as useful tools for chronic pruritus and psychodermatologic management. Their possible anti-inflammatory and wound-healing effects remain interesting, but human evidence is still limited. For now, beyond itch, the evidence is promising but not yet solid enough to completely change current practices. Moreover, patient education plays a key role in improving treatment adherence and enabling the early recognition of cutaneous adverse reactions, which may help to reduce the severity of these events and facilitate timely clinical intervention.

## 6. Antipsychotics

Psychotropic compounds such as lithium and valproate are frequently utilized in therapies for schizophrenia, seizures, and bipolar disorder. Antipsychotics function by blocking mainly dopaminergic D2 receptors in the brain or acting as antagonists of the 5-HT2A and 5-HT2C serotonin receptors [160].

Various studies have reported that these compounds can cause cutaneous adverse reactions, including exanthematous eruptions, skin pigmentation changes, photosensitivity, urticaria, and pruritus [161]. For instance, lithium has been linked to dermatological side effects such as psoriasis, acne, and folliculitis, while valproate administration has been associated with hair loss [162,163]. However, the impact of valproic acid (the active profile of valproate) differs significantly depending on whether it is administered systemically or topically. When ingested orally, it can induce diffuse, non-scarring alopecia, showing a clear dependence on the administered dose [162]. Conversely, local topical administration of valproic acid in in vivo assays demonstrates entirely opposite effects, promoting hair regeneration and wound healing [162,164]. Furthermore, in vitro assays utilizing HaCaT human keratinocytes have shown that a concentration of 100 µM significantly enhances cellular motility and migration [164]. Clinically, the topical application of valproate sprays at concentrations of 7.2% and 8.3% has been shown to improve hair regeneration and final hair density [162].

Regarding lithium, its systemic use is associated with adverse cutaneous conditions, most notably the exacerbation or de novo induction of ordinary, pustular, or palmoplantar psoriasis [163]. In vitro, lithium acts by reducing intracellular levels of inositol and cyclic adenosine monophosphate (cAMP), which alters calcium homeostasis and subsequently inhibits differentiation while inducing hyperproliferation of keratinocytes. Additionally, lithium exposure has been linked to neutrophil activation and the release of proinflammatory cytokines, such as TGF-aα, IFN-ϒ, and IL-2 [165].

Clozapine has also been associated with cutaneous adverse reactions, including hypersensitivity manifestations and, in rare cases, severe drug reactions [151,166].

Although a clear relationship exists between the skin and these psychotropic substances, further studies must be conducted to fully explore the potential therapeutic applications of these compounds in dermatology. To provide a clinically oriented overview, the main dermatological applications, benefits, and safety considerations of psychotropic compounds are summarized in Table 2.

## 7. Conclusions

Although unexpected, skin is emerging as a highly promising target for psychotropic compounds. As seen in this review, cannabinoids, psychedelics, and antidepressants can interact with cutaneous neuroimmune and neuroendocrine pathways, thus influencing inflammation, pruritus, barrier function, cellular senescence, and tissue repair through different but overlapping mechanisms. The presence of a cannabinoid system in the skin with a range of biological effects represents an interesting therapeutic target for different applications such as atopic dermatitis, psoriasis or wound healing. However, research is still in the process of characterizing these pathways and, above all, trying to overcome the limitation of improving their penetration into the skin, as most compounds remain confined to the superficial epidermal layers.

While cannabinoids currently hold the strongest translational potential, particularly in inflammatory and barrier-related disorders, psychedelics represent an exciting frontier in regenerative and anti-aging dermatology, and antidepressants continue to demonstrate clear value in psychodermatology and chronic pruritus, with additional promise in wound healing. Nevertheless, most evidence remains preclinical, and significant challenges persist regarding formulation, safety, regulatory approval, and clinical validation. Future research should focus on rigorous human trials, optimized topical delivery systems, and a deeper understanding of skin-specific pharmacology. Additionally, interindividual variability, including genetic predisposition, may influence responses to psychotropic compounds across different classes, highlighting the need for more personalized approaches in dermatological therapies.

Additionally, emerging tools such as artificial intelligence are increasingly being applied in dermatology, where they support the analysis of clinical and imaging data and improve diagnostic accuracy. Furthermore, recent advances suggest that AI-based models may contribute to predicting adverse drug reactions and optimizing therapeutic decision making, enabling earlier detection and more personalized risk assessment [167,168,169,170].

In addition, patient education represents a key component in the clinical use of psychotropic compounds, as it may improve treatment adherence and facilitate early recognition of adverse cutaneous reactions across different drug classes.

Ultimately, the convergence of dermatology, neuroscience, and psychopharmacology may open new innovative therapies, transforming compounds once viewed solely through a psychiatric lens into valuable tools for cutaneous medicine.

## Figures and Tables

**Table 1:** Molecular targets, signaling pathways, and cutaneous effects of major classes of psychotropic compounds. All abbreviations can be found in the Abbreviations section after the Conclusions.

Class	Main Compounds	Molecular Targets	Signaling Pathways	Cutaneous Effects
Cannabinoids	THC, CBD, CBG	CB1, CB2, TRPV1	NF-κB, MAPK, ECS	Anti-inflammatory, antipruritic, barrier modulation, wound repair
Psychedelics	Psilocin, DMT, LSD	5-HT2A, SIGMAR1	PI3K/Akt, Ca^2+^ signaling, serotonergic pathways	Anti-inflammatory, immune modulation, senescence regulation
Antidepressants	Fluoxetine, sertraline, doxepin	SERT, 5-HT receptors, MRGPRX2	cAMP, serotonergic signaling	Antipruritic, immunomodulatory, wound healing
Antipsychotics	Lithium, Valproate	D2, 5-HT2A, 5-HT2C	PLC/PKC, Ca^2+^ signaling, serotonergic pathways	Wound healing, hair regeneration

**Table 2:** Clinical considerations and safety of psychotropic compounds in dermatology.

Class	Main Dermatological Uses	Potential Benefits	Main Cutaneous Adverse Effects	Clinical Considerations
Cannabinoids	Atopic dermatitis, psoriasis, pruritus, wound healing	Anti-inflammatory, antipruritic, barrier repair, immunomodulation	Mild irritation (topical), limited systemic data	Promising but limited clinical evidence; formulation and penetration remain challenges
Psychedelics	Experimental (anti aging, immune modulation)	Anti inflammation, senescence modulation	Unknown in clinical dermatology; potential irritation	Mostly preclinical evidence; strong regulatory limitations
Antidepressants	Chronic pruritus, psychodermatologic disorders	Anti-pruritic, immunomodulatory, potential wound healing	Pruritus, urticaria, photosensitivity, rare severe reactions	Strongest clinical evidence for pruritus; monitor adverse effects
Antipsychotics	Limited	Possible wound healing, hair regeneration	Psoriasis (lithium), alopecia (valproate), hypersensitivity reactions	Effects are drug- and context-dependent; clinical use remains limited

## Data Availability

No new data were created or analyzed in this study. Data sharing is not applicable to this article.

## Abbreviations

The following abbreviations are used in this manuscript:

1-NPZ	1-naphthylpiperazine
5-HT	5-Hydroxytryptamine
ACEA	Arachidonyl-2′-chloroethylamide
ACTH	Adrenocorticotropic hormone
BCP	b-Caryophyllene
CAM	Cell adhesion molecule
cAMP	Cyclic adenosine monophosphate
CB	Cannabinoid receptor
CBC	Cannabichromene
CBD	Cannabidiol
CBG	Cannabigerol
CHS	Contact hypersensitivity
CPD	Cyclobutene pyrimidine dimer
CRH	Corticotropin releasing hormone
DiMiLs	Drug-in-micelles-in-liposomes
DMT	N,N-dimethyltryptamine
EB	Epidermolysis bullosa
ECS	Endocannabinoid system
FAAH	Fatty acid amide hydrolase
GH	Growth hormone
HF	Hair follicle
HGHL	High-glucose high-lipid
HPT	Hypothalamus pituitary thyroid
IFN	Interferon
IL	Interleukin
LPS	Lipopolysaccharide
LSD	Lisergic acid diethylamide
MAGL	Monoacylglycerol lipase
MAPK	Mitogen activated protein kinase
NF-KB	Nuclear factor kappa B
NLC	Nanostructured lipid carrier
PGE	Prostaglandin E
PI3K	Phosphatidylinositol 3-kinase
PKC	Protein kinase C
PLC	Phospholypase C
PPAR	Peroxisome proliferator–activated receptor
pRB	Protein retinoblastoma
ROS	Reactive oxigene species
SERT	Serotonin transporter
SLN	Solid lipid nanoparticle
SSRI	Selective serotonin reuptake inhibitor
TCAs	Tricyclic antidepressants
TCBM	Topical cannabis-based medication
TGFb1	Transforming growth factor b1
THC	Tetrahydrocannabinol
TNF	Tumor Necrosis factor
TRH	Thyrotropin releasing hormone
TRPV	Transient receptor potential cation channels
TSH	Thyroid-stimulating hormone
